# International Dental Congress

**Published:** 1887-03

**Authors:** Geo. A. Mills

**Affiliations:** Secretary of the Conference


### INTERNATIONAL DENTAL CONGRESS.

A conference of dentists who were in attendance at the eighteenth anniversary of the First District Society of New York was called at the Sturtevant House to consider the feasibility and advisability of taking steps for the calling of an International Dental Congress at some future date. Thirty-five dentists were in attendance, among whom were Drs. Northrop, Dwinelle, Littig, Walker, Perry, Carr, Kingsley, and Mills, of New York; Keech, Coyle, Winder, and Waters, of Baltimore; Hunt, of Washington; Darby, Truman, and Peirce, of Philadelphia; Brophy and Harlan, of Chicago; Shumway, of Massachusetts; A. P. Dickinson, of Iowa City; Watkins, Meeker, Brown, Levy, and others, of New Jersey; Bartholomew, of Springfield, Mass., and others whose names escaped the secretary.

The meeting was called to order and elected Dr. W. H. Dwinelle, chairman, and Dr. Geo. A. Mills, secretary.

Dr. Kingsley opened the discussion by some remarks in which he considered the advisability of taking steps for organizing a meeting to be called an International Dental Congress, and he offered the following resolution :

*Resolved,* That in the opinion of the conference the interests of the dental profession throughout the world will be advanced by an International Dental Congress.

A discussion then followed, in which Drs. Northrop, Truman, Brophy, Hunt, Winder, Keech, Kingsley, and the chairman took part. There was no difference of opinion as to the advisability of holding such a congress at some future time, and the years 1888, 1889, and 1891 were severally suggested and considered. The year 1890 was out of the question, because of another International Medical Congress to be held in that year. As to the time, no decision was reached. The resolution was carried unanimously.

A second resolution was offered, viz.:

*Resolved,* That the following named gentlemen constitute a Committee of Temporary Organization, whose duty it shall be to make such a plan for a permanent organization as shall in their estimation best call out universal support.

This was also discussed and carried. The committee named is Drs. Dwinelle, Northrop, Walker, Kingsley, Winder, Hunt, Coyle, Brophy, Levy, Meeker, Southworth, Frank French, Truman, Peirce, and Flagg.

A third resolution was offered and carried, viz.:

*Resolved,* That this committee be empowered to fill vacancies and enlarge its numbers at their discretion.

This conference was amicable in a large sense, yet there was a free interchange of opinion, while all did not think alike in all things. Wise measures were strongly advocated, so that it should not appear that there was any disposition to place obstructions in the way of any movement that sought the best good of all.

The meeting adjourned subject to the call of the chair.

GEO. A. MILLS,
*Secretary of the Conference.*

